# Pathogenic role of the staphylococcal accessory gene regulator quorum sensing system in atopic dermatitis

**DOI:** 10.3389/fcimb.2023.1178650

**Published:** 2023-04-14

**Authors:** Masakazu Tamai, Yuriko Yamazaki, Tomoka Ito, Seitaro Nakagawa, Yuumi Nakamura

**Affiliations:** ^1^ Department of Dermatology, Osaka University Graduate School of Medicine, Osaka, Japan; ^2^ Cutaneous Allergy and Host Defense, Immunology Frontier Research Center, Osaka University, Osaka, Japan; ^3^ Department of Pathology and Rogel Cancer Center, University of Michigan Medical School, Ann Arbor, MI, United States

**Keywords:** atopic dermatits, *Staphycoccus aureus*, Agr, quorum sensing, Th2, IL-17, dysbiosis

## Abstract

The skin is home to various bacteria, archaea, fungi, and viruses, collectively referred to as the skin microbiota. Patients with certain skin diseases reportedly have unique skin “dysbiosis,” a condition involving imbalanced microbiota, suggesting that dysbiosis in the skin may be either causal or a consequence of specific skin diseases. Atopic dermatitis (AD) is the most common allergic skin disease that affects 15-20% of children and 2-10% of adults worldwide. Both intrinsic genetic factors, such as susceptibility to type 2 inflammation or skin barrier dysfunction, and extrinsic environmental factors, such as air pollen and skin microbiota, contribute to AD. *Staphylococcus aureus*, which does not often colonize the skin of healthy individuals, is commonly identified in the lesional skin of patients with AD and is correlated with the disease flare. However, the role of *S. aureus* in the pathogenesis of AD has not been elucidated. Here, we discuss the pathological behavior of *S. aureus*, focusing on accessory gene regulator (Agr) quorum sensing, which is a fundamental bacterial cell-to-cell interaction mechanism that affects the behavior of *S. aureus* and other members of the microbial community. Importantly, beyond bacteria-bacteria interactions, the Agr quorum sensing system also regulates various virulence factors, which induce type 2 and IL-17-dependent skin inflammation in the host. Furthermore, the colonization of Agr-positive *S. aureus* in early life accelerates the development of pediatric AD. Finally, we aim to highlight the current efforts to establish novel therapeutic methods to ameliorate or prevent AD through Agr-targeted intervention.

## Introduction

The skin is the largest organ of the human body and plays several crucial roles in maintaining human health. It is a physical barrier that protects the host from bacterial invasion and harmful external environmental factors (Langan et al., 2020). The skin contains a diverse microbial population comprising bacteria, fungi, and viruses, referred to as the microbiota. Recent studies have revealed a correlation between the microbiota and various diseases, including atopic dermatitis (AD) (Cani and Jordan, 2018; Paller et al., 2019). AD is a chronic inflammatory skin disorder that affects a significant proportion of the population in industrialized nations, with a prevalence of 20% in children and approximately 5% in adults in high-income countries (Langan et al., 2020). AD is clinically characterized by red, dry and itchy skin lesion. Sleep disturbance due to severe itching is one of the major symptoms of AD, and it can significantly impair patients’ and their caregivers’ quality of life and social productivity (Chang and Chiang, 2018).

AD is a complex disease characterized by a Th2 cell-mediated immune response, which is the central mechanism underlying its pathogenesis (Gittler et al., 2012). In acute phase of AD, the skin barrier is disrupted and allergens are presented to antigen-presenting cells bearing specific IgE to enhance Th2 response (Werfel et al., 2016). Thymic stromal lymphopoietin (TSLP) and interleukins released from keratinocytes stimulate Th2 cell and Langerhans cell development. Th2 cell subsequently produces cytokines, Interleukin (IL)-4, IL-13, and IL-31, which directly activate sensory nerves and promote pruritus (Sonkoly et al., 2006). At the same time, IL-4, IL-13 induces inflammatory cytokines and decreases keratinocytes production of filaggrin, loricrin, and involucrin, further decreasing the barrier function (Kim et al., 2008; Werfel et al., 2016). In contrast, in the chronic phase, the expression of the type-1 cytokine such as interferon-gamma are heightened (Werfel et al., 1996; Tsoi et al., 2020). It is also linked to the activities of various T cell subsets, such as the Th22, Th17/IL-23, and Th1 cytokine pathways (Gittler et al., 2012). Although various immune dysfunctions related to AD have been widely investigated, the underlying causes of aberrant immune responses remain unclear. The development of AD is believed to result from the interplay between environmental factors, as represented by the hygiene hypothesis, and the host’s genetic background (Flohr et al., 2005). One such environmental factor is skin dysbiosis. Our group has made important discoveries on the relationship between AD and *Staphylococcus. aureus*. (Nakamura et al., 2013; Nakagawa et al., 2017; Nakamura et al., 2020) Here, we review the relationship between skin dysbiosis and AD, focusing on the accessory gene regulator (Agr) quorum sensing mechanism, which plays an essential role in the virulence of *S. aureus*.

## The role of the skin microbiota in host immunity

The surface area of an adult individual’s skin including not only exposed interfollicular epithelium but the epithelial lining of hair follicles, eccrine ducts, apocrine ducts, and sebaceous glands encompasses approximately at least 30 square meters and harbors an estimated 40 distinct bacterial strains, with a concentration of 10^6^ bacteria per square centimeter (Belkaid and Segre, 2014; Gallo, 2017). The skin microbiota of neonates is acquired during parturition and varies depending on the delivery mode. Neonates born via vaginal delivery possess skin microbiota similar to the maternal vaginal microbiota, whereas those born via cesarean section exhibit skin microbiota similar to the maternal skin microbiota (Mueller et al., 2015). As individuals age, skin microbial composition gradually shifts. During puberty, the skin microbiota undergoes significant changes, particularly in areas where the sebaceous glands develop, leading to a stable condition of skin microbial communities in adults (Oh et al., 2012; Si et al., 2015). The normal skin microbiota obtained through this process plays a vital role in the host’s immune system (Chen et al., 2018). Human skin acquires memory regulatory T cells progressively from the neonatal to adult stages, which contribute to developing immune tolerance against commensal bacteria. One study showed that the colonization of *Staphylococcus epidermidis*, a representative normal skin microbiota species, during the neonatal period causes CD4+ T regulatory cells to migrate to the skin and generate tolerance towards commensals in mice (Naik et al., 2015; Scharschmidt et al., 2015). In contrast, IL-17A+ CD8+ T cells act as a defensive mechanism against pathogenic microorganisms (Scharschmidt et al., 2015). Additionally, in adult mice, *S. epidermidis* on the skin stimulates the migration of IL-17A+ CD8+ T cells, enhancing innate immunity against pathogens such as *Candida albicans* and *Leishmania major* (Belkaid et al., 2002; Naik et al., 2015). Among staphylococci, *S. epidermidis*, and *Staphylococcus hominis* are representative commensal species of coagulase-negative staphylococci in human skin (Byrd et al., 2018), and produce antimicrobial peptides that target pathological bacteria, such as *S. aureus*. Commensal staphylococci also stimulate the host to produce antimicrobial peptides, including β-defensin-2, which aid in eliminating pathogenic microorganisms (Nagy et al., 2006; Gallo and Hooper, 2012; Zipperer et al., 2016; Nakatsuji et al., 2017). Thus, the resident bacterial flora plays an essential role in maintaining the balance of the host skin immune system in a healthy state (Sanchez Rodriguez et al., 2014; Naik et al., 2015).

## Skin microbiota and Staphylococcus virulence factors in AD

An initial study, which was conducted in the 1960s, revealed that individuals with AD had the presence of *S. aureus* in their affected skin (Selwyn, 1963). In 1974, a significant amount of *S. aureus* was found on the skin of patients with AD, even in cases with no visible signs of infection (Leyden et al., 1974). Recent studies have revealed that disturbances in normal microbiota or dysbiosis can be an underlying cause or consequence of various diseases (Sanford and Gallo, 2013). Patients with AD typically exhibit a distinctive composition of skin microbiota, characterized by reduced richness, depletion of some commensal bacteria, and enrichment of staphylococci (Kong et al., 2012; Tay et al., 2021). Specifically, skin lesions of patients with AD often show colonization by pathogenic *S. aureus*, which does not normally colonize the skin of healthy individuals, and abnormal growth of *S. epidermidis* (Kong et al., 2012; Byrd et al., 2017). The percentage of individuals carrying *S. aureus* in their lesional skin area varied between reports. According to a meta-analysis of 95 observational studies among patients with AD, the prevalence of *S. aureus* colonization on lesional skin was 70%. In contrast, it was only 39% in non-lesional or healthy control skin (Totté et al., 2016). Additionally, the prevalence of *S. aureus* colonization increases with disease severity (Totté et al., 2016). Furthermore, real-time PCR has revealed that the density of *S. aureus* correlates with disease severity (Tauber et al., 2016).

Presumably, molecules of *S. aureus*, such as α-hemolysin (Hla) (Travers et al., 2003), lipoteichoic acid (LTA) (Travers et al., 2010), peptidoglycan (PGN) (Matsui and Nishikawa, 2012), and staphylococcal protein A (SPA), are involved in inflammatory reactions of the skin. LTA and PGN from *S. aureus* have been reported to induce inflammatory responses in the skin as Toll-like receptor ligands (Travers et al., 2010; Matsui and Nishikawa, 2012). However, these molecules are also present in other commensal skin bacteria, and their specificity has not yet been demonstrated. Hla triggers potassium efflux upon insertion into the membrane of human keratinocytes, and topical application of Hla induces skin barrier disruption and inflammation in SKH-HR1 hairless mice (Travers et al., 2003; Hong et al., 2014). Although SPA can disrupt opsonization and phagocytosis by the host immune system and trigger IL-18 production by keratinocytes (Spika et al., 1981), topical application of SPA did not induce skin inflammation in a mouse model (Nakano et al., 2003). Superantigens (SAgs) such as toxic shock syndrome toxin-1 (TSST-1) and staphylococcal enterotoxins bind to major histocompatibility class II (MHC II) molecules on the surface of antigen-presenting cells and T cell receptors on T cells, bypassing the constraint of antigenic peptide presentation. Although these SAgs can cause toxic shock syndrome in humans, their effect on AD severity is not significant (Kozman et al., 2010; Rojo et al., 2014). However, these previous studies did not analyze the role of *S. aureus* pathogenic factors in the skin of mice epicutaneously colonized by live *S. aureus*. Therefore, whether these factors contribute to AD pathogenesis on the skin surface remains unclear.

In addition to *S. aureus, S. epidermidis* has also been reported as a pathogenic bacterium in AD. A previous report has mentioned that an increasing number of *S. epidermidis* in the lesional skin of patients with AD produce EcpA, which contributes to dermatitis (Cau et al., 2021). In contrast, individual strains of *S. epidermidis* can inhibit biofilm formation by *S. aureus* through the production of Esp, a serine protease present in normal microbiota (Iwase et al., 2010). Thus, the pathogenicity of *S. epidermidis* in AD is not as well established as that of *S. aureus* and remains debatable.

In summary, differences in the microbiota exist between the normal skin and the skin of patients with AD. Dysbiosis of the lesional skin, characterized by a shift in the proportion of staphylococci and an increase in pathogenic strains, contributes to the exacerbation of AD.

## *Staphycoccus aureus* Agr quorum sensing in AD pathogenesis

**Figure 1:**
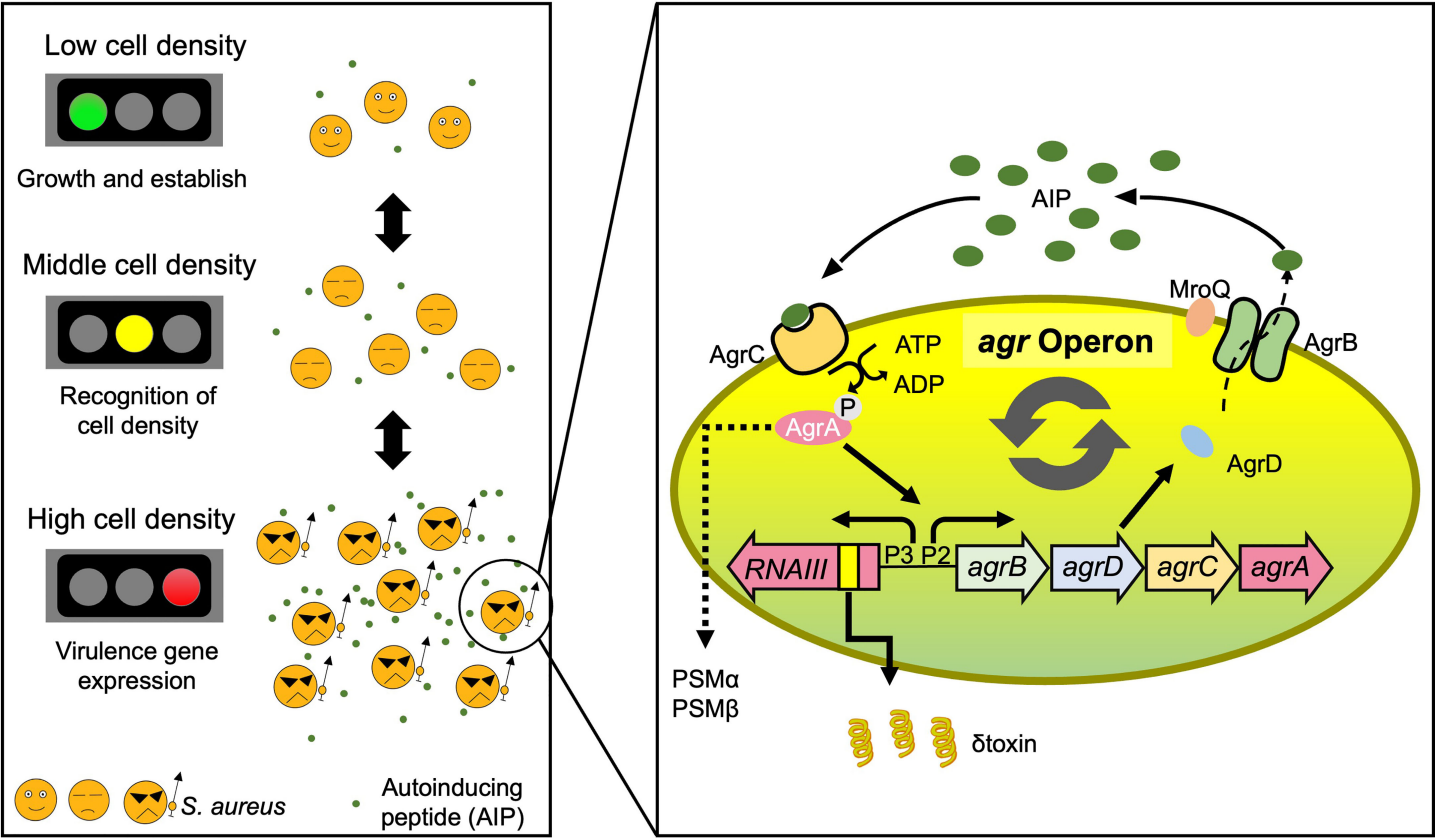
A proposed model for the role of *S. aureus* QS-regulated Agr virulence. *S. aureus* secretes AIP, thereby informing each other of their own cell population density, and when the concentration exceeds a certain threshold, they turn on virulence gene expression. The *agr* operon is activated in a positive feedback manner once QS is triggered by AIP. The AIPs of groups 1, 2, and likely 4 are reported to be exported by AgrB and trimmed at the N-terminus by the non-Agr-encoded membrane-located protease MroQ. The Agr-QS system controls the expression of several virulence factors in *S. aureus*. AD, atopic dermatitis; QS, quorum sensing; AIP, auto inducing peptide.

Quorum Sensing (QS) is the ability of bacteria to detect the cell population density. When the bacterial population is high, they are at risk of nutrient scarcity. Thus, bacteria change their behavior by regulating the production of various genes and substances to obtain more nutrients from the host or eliminate bacteria with similar nutritional requirements. *S. aureus* possesses an auto-regulatory operon, Agr system, as a QS function. *S. aureus*, similar to a pheromone, secretes an exocrine auto-inducing peptide (AIP) to communicate with and sense its species. When the population density is high, and AIP reaches the threshold for activating the transmembrane receptor (AgrC) on the cell surface, autophosphorylation of the histidine kinase AgrC is triggered. AgrC binds to AgrA, a DNA-binding response regulator, and activates AgrA via phosphorylation. AgrA directly binds to *agr* region to activate two bidirectional promoters, P2 and P3. Under the P2 promoter regulation, *agrB* encodes the AIP transporter in the extracellular space and *agrD*, encoding the precursor of AIP, and *agrC* and *agrA*. There are four subcategories of Agr, exhibiting distinctions in the amino acid sequence of the mature autoinducing peptide (AIP) (Ji et al., 1997). Recently, the AIPs of groups 1, 2, and likely 4 are reported to be exported by AgrB and trimmed at the N-terminus by the non-Agr-encoded membrane-located protease MroQ (Stock et al., 2022; Zhao et al., 2022). Thus, the *agr* operon is activated in a positive-feedback manner once QS is triggered. In contrast, the P3 promoter regulates various toxins via RNAIII, a regulatory RNA (Le and Otto, 2015) (**Figure 1**).

The phenol-soluble modulin (PSM) family is a major virulence factor of Staphylococcus and is regulated by the Agr-QS system (Wang et al., 2007; Otto, 2014). *S. aureus* possesses four PSMα peptides (PSMα1–4), two PSMβ peptides (PSMβ1 and PSMβ2), and *δ*-toxin (PSMγ). The *psmα* and *psmβ* operons are directly regulated by AgrA, while *δ*-toxin is encoded within the *RNAIII* operon and regulated under the P3 promoter (Le and Otto, 2015) (**Figure 1**).

*S. aureus* uses Agr-QS to behave differently in various situations. During chronic infections, *S. aureus* forms biofilms and exhibits strong antibiotic resistance when *agr* is suppressed (Schilcher and Horswill, 2020). However, as the biofilm matures, Agr-QS is turned on, and PSM production enables bacterial cells to detach from the biofilm and spread (Boles and Horswill, 2008). Additionally, α-type PSMs, particularly PSMα3, strongly induce neutrophil chemotaxis and Ca^2+^ flux, which are highly toxic to neutrophils (Wang et al., 2007; Otto, 2014). Using community-acquired methicillin-resistant *S. aureus* (CA-MRSA), the PSMα deletion strain, but not the other PSM deletion strains, had a significantly decreased ability to cause skin abscess formations in mice (Wang et al., 2007). These results suggest that PSMα, via Agr-QS activation, may contribute to developing CA-MRSA skin and soft tissue infections (Wang et al., 2007; Otto, 2014). *S. aureus* utilizes Agr-QS to control the expression of virulence factors during growth (Miller and Bassler, 2001). We have previously reported that Agr-QS in *S. aureus* plays a crucial role in skin adaptation and AD pathogenesis (Nakamura et al., 2013; Nakagawa et al., 2017). In the following sections, we elaborate on the pathogenic role of *S. aureus* Agr-QS in AD.

### *δ*-toxin

**Figure 2:**
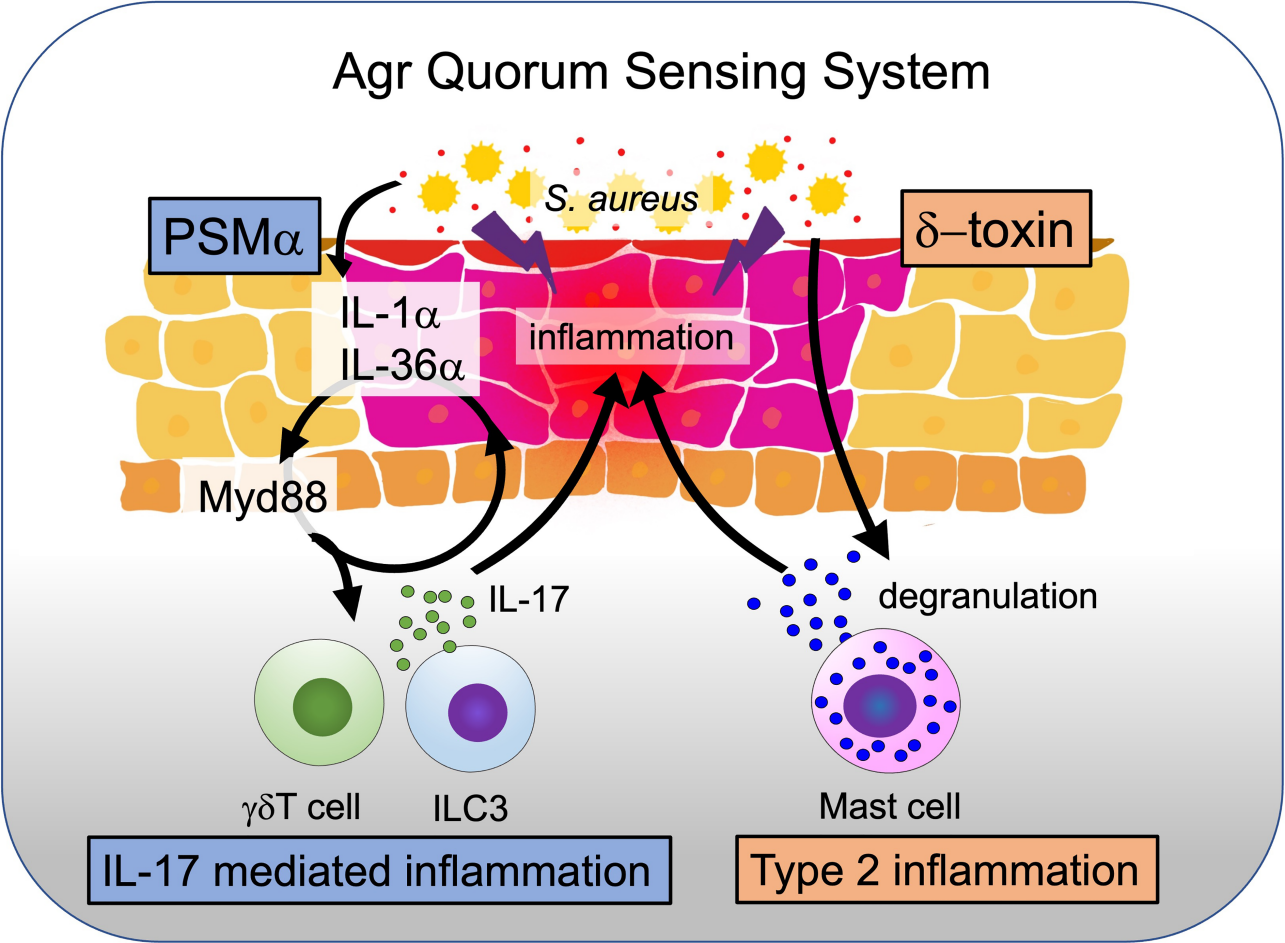
How δ-toxin and PSMα contribute to exacerbating AD. When *S. aureus* colonizes the skin surface and triggers the quorum sensing system, virulence factors such as δ-toxin and PSMα are produced. δ-toxin causes mast cell degranulation and triggers type II inflammation, while PSMα causes the release of IL-1α and IL-36α alarmin from keratinocytes and triggers IL17-mediated dermatitis.

Although the severity of AD is associated with the presence of *S. aureus*, the precise role of this bacterium in disease pathophysiology remained unclear (White and Noble, 1986). This may be because, despite the presence of bacteria in patients with AD on the outermost layer of the skin, the stratum corneum, the previous mouse models examining the pathogenicity of *S. aureus* in the skin utilized bacterial injection into the subcutaneous tissue (Yoshioka et al., 2003; Bin et al., 2012; Malachowa et al., 2013). To address this issue, we adapted an ovalbumin (OVA) epicutaneous sensitization model and established an epicutaneous *S. aureus* inoculation mouse model (Spergel et al., 1998; Nakamura et al., 2013). Using this model, we revealed that the expression of *RNAIII*, which is indicative of the positive expression of Agr-QS, was observed four days after epicutaneous inoculation of live *S. aureus*. Mice colonized with *S. aureus* developed severe dermatitis on day 7. Because we identified δ-toxin from *S. aureus* via activation of Agr-QS strongly inducing mast cell degranulation *in vitro*, we compared *S. aureus* wild-type and δ-toxin deficient strains in this model (Nakamura et al., 2013). Mice colonized with *S. aureus* lacking δ-toxin showed significantly reduced skin inflammation compared to wild-type colonized mice. In addition, precolonization with wild-type *S. aureus*, but not with δ-toxin deficient *S. aureus*, enhanced the production of OVA-specific IgE in OVA-exposed mice. These responses are abolished in mast cell-deficient mice. Thus, we found that Th2-type dermatitis was induced through mast cell degranulation triggered by δ-toxin under Agr-QS expression. Furthermore, we analyzed *RNAIII* expression in wash fluid obtained from the lesional and non-lesional skin of patients with AD and found that *RNAIII* expression was only upregulated in AD lesional skin, indicating the involvement of Agr-QS in AD exacerbation in humans (Nakamura et al., 2013; Nakagawa et al., 2017) (**Figure 2**).

### PSMα

Our research group further elucidated how PSMα acts on keratinocytes to induce dermatitis (Nakagawa et al., 2017). Using primary keratinocytes, we found that PSMα induces epidermal keratinocyte cell death and stimulates the release and secretion of the alarmins IL-1α and IL-36α via Myd88, a signaling adaptor of IL-1 receptor (IL-1R) and IL-36 receptor (IL-36R). Furthermore, we performed epicutaneous *S. aureus* inoculation using IL-1R deficient (*Ilr1*-/-) mice and an IL-36R neutralizing antibody. Only *Ilr1*-/- mice treated with anti-IL-36R antibody, but not *Ilr1*-/- or IL-36R antibody-treated wild-type mice, showed dramatically suppressed dermatitis compared to wild-type mice, indicating that both IL-1 and IL-36 are important for the induction of skin inflammation in epidermal *S. aureus* inoculation. Additionally, IL-1R signaling plays an important role in Th17 cell differentiation (Chung et al., 2009). Flow cytometry analysis of cells from skin lesions in a mouse model of *S. aureus* epidermal inoculation showed a dramatic increase in IL-17A-producing cells and a small increase in cells producing IL-17F and IL-22, suggesting that IL-1R signaling is involved in Th17 cell differentiation and the subsequent induction of skin inflammation. When *S. aureus* was inoculated on IL-17A and IL-17F double-knockout mice lacking IL-17 signaling, skin inflammation was attenuated compared to that in wild-type mice, whereas the number of *S. aureus* grown on the skin was comparable. To identify the molecule responsible for *S. aureus* that releases alarmin from keratinocytes, we stimulated keratinocytes with culture supernatants of *S. aureus* wild-type and various toxin-deficient strains. The culture supernatant of the *S. aureus* wild-type strain, but not that of the PSMα deficient strain, strongly enhanced the release of alarmins from keratinocytes. PSMα was also essential for inducing IL-17-dependent dermatitis via the release of alarmins in our epicutaneous *S. aureus* inoculation model (Nakagawa et al., 2017). A previous report also demonstrated that IL-17 had been reported to be involved in the pathogenesis of AD in infancy (Esaki et al., 2016). The induction of IL-17-dependent dermatitis by PSMα suggests that the activated Agr-QS of *S. aureus* on the skin may be responsible for AD development (**Figure 2**).

### Other molecules

Previously, it was reported that MroQ is an important mediator of *S. aureus* pathogenesis in strains harboring a type I and type II Agr variant (Zhao et al., 2022). MroQ functions as a membrane protease regulator of Agr and is also an essential component of AIP biosynthesis (Zhao et al., 2022). In a mouse intradermal infection model, a *mroQ* mutant of Agr type I showed a dramatic reduction in abscess formation (Stock et al., 2022). However, the importance of MroQ in an epicutaneous model has not been investigated, and a *mroQ* mutant was not found in infant skin isolates from an AD infant cohort study. Therefore, the role of MroQ in the pathogenesis of AD remains unclear. Another report mentioned that eight of 10 AD skin-adapted *S. aureus* strains harbored mutations in *fumC* and other metabolic genes and stimulate inflammatory responses in keratinocytes *in vitro* (Acker et al., 2019). Although an AD-associated mutant strain was also only tested in intradermal injection mouse model, a skin-adapted isolate failed to generate protection from a secondary infectious challenge (Acker et al., 2019).

## Agr quorum sensing drives *Staphycoccus aureus* skin colonization in AD infants

**Figure 3:**
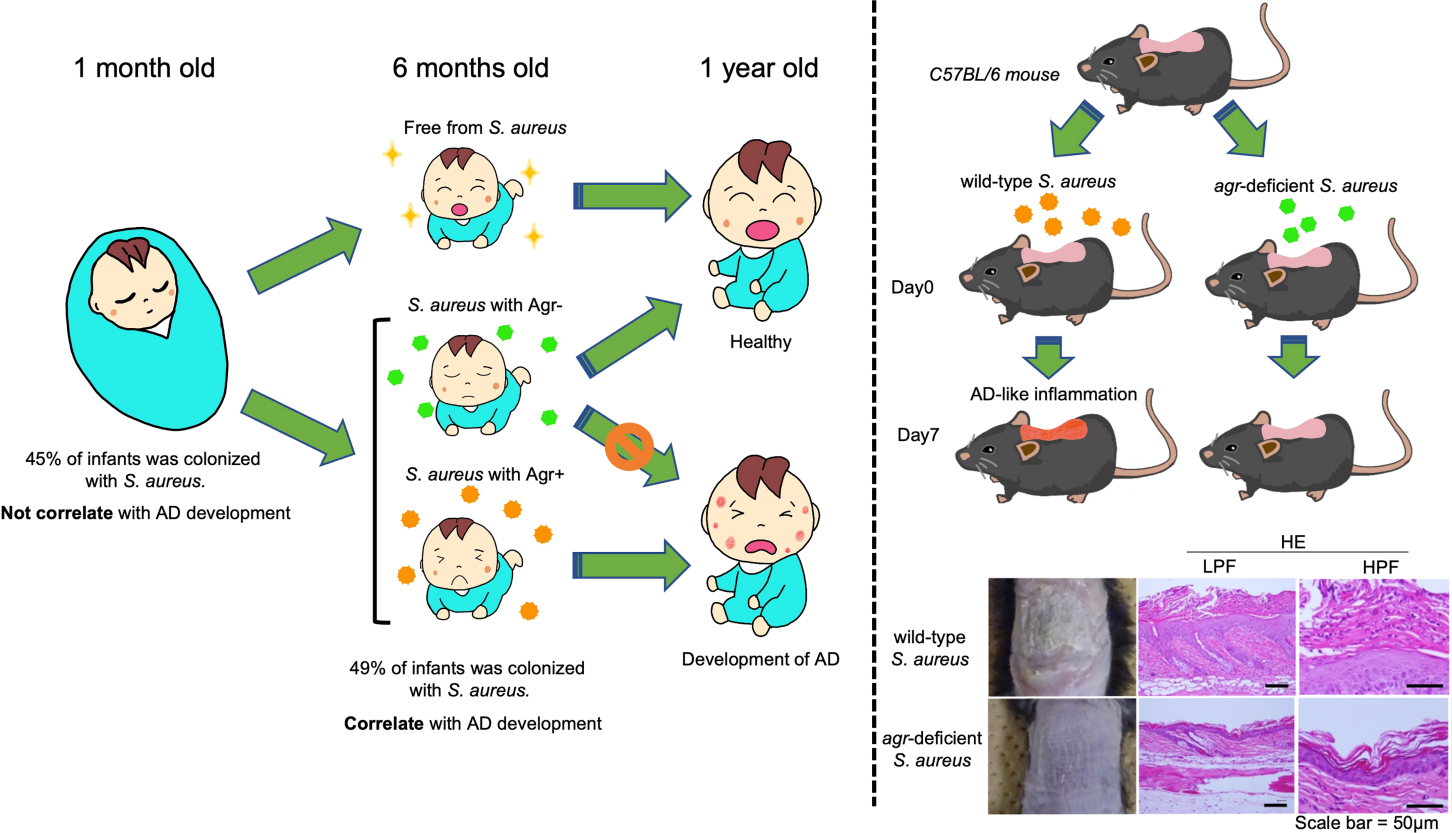
Agr quorum sensing drives *S. aureus* skin colonization in AD infants. The risk of developing AD was significantly higher in infants who were colonized with *S. aureus* at six months. The *agr* mutations of *S. aureus* occurred in strains from those infants who did not develop AD, while no *agr* mutations of *S. aureus* were identified in the infants who did develop AD. Epicutaneous *S. aureus* colonization mouse model revealed that the Agr-QS system plays a critical role in the development of AD-like inflammation. The photos and pathological images of mice were adapted from (Nakamura et al., 2020). HE, Hematoxylin-Eosin Stain; HPF, high power field; LPF low power field.

Examination of the virulence factors modulated downstream of Agr-QS indicated that *S. aureus* exacerbates skin inflammation in AD. However, it remains unclear why *S. aureus* is disproportionately present in AD skin and rarely found in healthy skin. Therefore, to elucidate the mechanism of *S. aureus* colonization before the onset of AD, we conducted a cohort study to chronologically obtain skin microbiota from the cheeks of 268 Japanese infants 1-6 months after birth (Nakamura et al., 2020). Approximately 45% of the infants were colonized with *S. aureus* at one month; however, the presence or absence of this colonization did not affect the risk of developing AD at one year of age. In contrast, the risk of developing AD was significantly higher in infants colonized with *S. aureus* at six months. We examined the genomic differences between *S. aureus* strains associated with AD and healthy skin. Using whole-genome sequencing of *S. aureus* strains, we discovered that loss-of-function mutations in the *agr* locus of *S. aureus* occurred in strains from infants who did not develop AD at 1 year of age. In contrast, no mutations in this region have been identified in infants with AD (Nakamura et al., 2020). Furthermore, our study, using an epicutaneous *S. aureus* colonization mouse model, revealed that the Agr-QS system plays a critical role in the epidermal colonization of *S. aureus* and the development of AD-like inflammation (Nakamura et al., 2020). These results suggest that the presence of a functioning Agr-QS system in *S. aureus* isolates from AD-associated infant skin contributes to skin colonization and AD development. In contrast, the absence of this system, due to a loss-of-function mutation in healthy infant skin, leads to the elimination of *S. aureus*. Thus, the maintenance of pathogenicity through the Agr-QS system correlates with *S. aureus* skin colonization and the emergence of AD (**Figure 3**). In contrast, another report mentioned that human skin grafts maintained on severe combined immunodeficiency disease (SCID) mice to follow the consequences of *S. aureus* infection showed that many more *agr* mutant USA300 MRSA strain are seen in the infected graft compared to its wild-type strain (Soong et al., 2015). Even though this may seem contrary to what we observed in AD infant cohort (Nakamura et al., 2020), when considering the background of an immunodeficiency in SCID mice, it may reflect the importance of host immunity in the colonization and pathogenesis of *S. aureus* on the skin (Soong et al., 2015).

## Interactions between *Staphycoccus aureus* and other bacterial species

The communication between bacteria in the human body has not been well understood until recently. Many researches are currently investigating the interactions of *S. aureus* with other bacterial species via QS and other molecules. Notably, recent work revealed that *S. epidermidis* can inhibit the formation of *S. aureus* biofilms by the secretion of Esp, a serine protease, which consequently inhibit the colonization of *S. aureus* upon epithelial surfaces (Iwase et al., 2010). In fact, in a Japanese epidemiological study analyzing *S. epidermidis* nasal colonization from 88 healthy volunteers, 44% of *S. epidermidis* strains were expressing Esp protease and those carrying “inhibitory type” *S. epidermidis* had significantly lower carriage rate of *S. aureus* in their nasal cavity (Iwase et al., 2010). After this discovery, other studies had also advanced the understanding of interactions between bacteria, particularly *S. aureus* and other bacteria species. For example, an *in vivo* study revealed that PSM γ and δ produced by *S. epidermidis* directly induce lipid vesicle leakage and exerted selective antimicrobial action against *S. aureus*. (Cogen et al., 2010) In another study, in 2016, a novel antimicrobial peptide named lugdunin, produced by *Staphylococcus lugdunensis*, was discovered and subsequently demonstrated to effectively inhibit the growth and colonization of *S. aureus*. (Zipperer et al., 2016) Whereas the presence of antibiotics pressure bacteria to adapt and eventually accelerate antimicrobial resistance in general, *S. aureus* incubated with lugdunin over a long period of time did not gain the resistance to lugdunin, letting this molecule a potential therapeutic drug to counter the drug-resistant *S. aureus*. (Zipperer et al., 2016) Moreover, a human microbiome-based study elucidated that coagulase-negative Staphylococcus (CoNS), which is predominant in the commensal flora, secrete lantibiotics, an antimicrobial peptides, to compete with *S. aureus* (Nakatsuji et al., 2017). Other researches are still investigating antimicrobial substances that do not act on commensal bacteria, but selectively on pathogenic bacteria such as *S. aureus and Escherichia coli*, and contribute to the stability of the resident microflora.

Other than the anti-microbial peptides, there is another attempt to inhibit *S.aureus* pathogenicity through inhibition of QS. These molecules with the capability of inhibiting the QS signals are named as Quorum Quenching (QQ) or quorum sensing inhibitor (QSI). Other staphylococci besides *S. aureus* also utilize AIPs for Agr-QS. Although these species share a common AIP structure, several AIP have different amino acid sequences. Thus, staphylococci with different types of AIP compete with each other, upregulating the expression of the Agr in bacteria of the same type of AIP while downregulating the expression of the *agr* in other staphylococci of different types of AIP (Ji et al., 1997; Otto et al., 2001). Most of the AIPs produced by non-*S. aureus* staphylococci have been found to act as quorum quenching against *S. aureus*. For example, *S. hominis* AIP inhibits the Agr of *S. aureus* and *S. epidermidis* (Severn et al., 2022). In addition to staphylococci, there are other bacterial genera that have the ability of QQ. In 2018, fengycins, a lipopeptide produced by Bacillus sp. were shown to inhibit *S. aureus* colonization of the nasal cavity and intestinal tract in humans and mice, through quorum quenching (Piewngam et al., 2018). Corynebacterium species, which are also important members of the human skin, seem to inhibit the *S. aureus* by quorum quenching as well. (Hardy et al., 2019) For example, *Corynebacterium striatum* has been reported to inhibit the production of toxins downstream of the quorum sensing mechanism, promote biofilm formation, and alter gene expression in *S. aureus* to make it behave more like a commensal bacteria than a pathogen (Ramsey et al., 2016). In summary, some coagulase-negative staphylococcus (CoNS) and Corynebacterium species help eliminate pathogenic bacteria by producing antimicrobial substances or through quorum quenching. In healthy skin, CoNS strains with such ability have been reported to be predominant, and the pathogenicity of *S. aureus* is more likely to demonstrate when these CoNS strains are absent or reduced (Nakatsuji et al., 2017; Williams et al., 2019). Thus, commensal bacteria cross-talk and form a microflora by interacting with each other through QS system and other signals.

## Agr quorum sensing is a potential therapeutic target for AD and *Staphycoccus aureus* skin infection

Based on previous research, it has been established that the Agr-QS in *S. aureus* is crucial for the bacterial colonization of the skin and the exacerbation of AD. Various approaches have been attempted to target *S. aureus* for the treatment of AD, including the use of antibiotics and disinfectants to eliminate *S. aureus*. In industrialized countries, including the United States, sodium hypochlorite bleach baths have been used for the treatment of AD to effectively eradicate *S. aureus* (Chopra et al., 2017). However, some reports have indicated that bleach baths do not significantly affect the commensal microbiota of the skin or *S. aureus* colonization (Gonzalez et al., 2016; Sawada et al., 2019). Moreover, although attempts have been made to eliminate *S. aureus* using antibiotics, such methods are ineffective and increase the risk of further dysbiosis and antimicrobial-resistant bacteria (Bath-Hextall et al., 2010).

Therefore, Agr-QS in *S. aureus*, rather than in the bacteria, has attracted the attention of researchers as a potential therapeutic target. Our group has also reported that Agr-QS inhibitors suppress the virulence of *S. aureus* and reduce skin inflammation in a mouse model of inflammatory skin disease variants (Baldry et al., 2018; Nakagawa et al., 2020). There have been numerous attempts to target AgrA and AgrC and other targets for quorum quenching, but rare *in vivo* experiments have shown efficacy in animal infection models, and the fact that it was found to be counterproductive biofilm-increasing effects makes it uncertain whether it can actually be used for treatment (Otto, 2023). There are also emerging approaches to using bacteria competing against *S. aureus* for bacteriotherapy. It has been reported that skin commensal CoNS can prevent skin infections of pathogenic *S. aureus* by exploiting its different AIP sequences (Paharik et al., 2017). Analysis of samples from patients has demonstrated that AIPs derived from CoNS, specifically *S. epidermidis*, inhibited the Agr-QS system of *S. aureus* and potentially ameliorated skin symptoms (Williams et al., 2019). A novel therapeutic approach utilizing competing bacteria to suppress the proliferation of *S. aureus* and Agr-QS is currently under development. In a phase I clinical trial, the efficacy of the ShA9 strain of *Staphylococcus hominis* was demonstrated by its application to the lesional skin of patients with AD for 1 week, which resulted in the suppression of *S. aureus*, correction of dysbiosis, and a certain degree of improvement in local eczema (Nakatsuji et al., 2021b). ShA9 also produces a lantibiotic that contributes to the suppression of *S. aureus*, although even the lantibiotic deletion mutant showed an effect by quorum quenching (Nakatsuji et al., 2021b). Additionally, in a small clinical trial, CoNS were collected from patients with AD and reintroduced into the lesion area of the same patient through autologous bacteriotherapy. This approach competitively inhibits and eradicates pathogenic *S. aureus*, leading to significant improvement in cutaneous symptoms (Nakatsuji et al., 2021a). Based on these findings, using commensal bacteria and compounds that effectively inhibit *S. aureus* Agr-QS shows great potential as a strategy for ameliorating AD symptoms.

## Conclusions

As discussed above, the Agr-QS is widely recognized as necessary for *S. aureus* colonization of the skin of patients with AD. The toxins produced by the Agr-QS can exacerbate the disease. However, whether atopic predisposition or bacterial colonization is the primary cause of AD development remains debatable. Further analysis is required to understand the mechanism that primarily controls the colonization and elimination of *S. aureus* from the skin. Targeting the mechanism of *S. aureus* Agr-QS in patients with AD may prevent the development and exacerbation of AD. This approach is completely different from conventional treatments for AD and is significant because it has the potential to prevent the development of AD and can lead to breakthroughs in the treatment of other *S. aureus* infections and, by extension, infections caused by other bacterial species.

## Author contributions

All authors listed have made a substantial, direct, and intellectual contribution to the work and approved it for publication.
